# Comparative transcriptome assembly and genome-guided profiling for *Brettanomyces bruxellensis* LAMAP2480 during *p-coumaric* acid stress

**DOI:** 10.1038/srep34304

**Published:** 2016-09-28

**Authors:** Liliana Godoy, Patricia Vera-Wolf, Claudio Martinez, Juan A. Ugalde, María Angélica Ganga

**Affiliations:** 1 Laboratorio de Microbiología Aplicada y Biotecnología, Departamento en Ciencia y Tecnología de los Alimentos, Facultad Tecnológica, Universidad de Santiago de Chile, Santiago, Chile; 2 Centro de Genética y Genómica, Facultad de Medicina, Clínica Alemana Universidad del Desarrollo, Santiago, Chile; 3 Centro de Estudios en Ciencia y Tecnología de Alimentos, Universidad de Santiago de Chile, Chile; 4 Programa de Genómica Microbiana, Facultad de Medicina, Clínica Alemana Universidad del Desarrollo, Santiago, Chile; 5 Millenium Nucleus for Fungal Integrative and Synthetic Biology (MN-FISB), Departamento en Ciencia y Tecnología de los Alimentos Universidad de Santiago de Chile, Santiago, Chile.

## Abstract

*Brettanomyces bruxellensis* has been described as the main contaminant yeast in wine production, due to its ability to convert the hydroxycinnamic acids naturally present in the grape phenolic derivatives, into volatile phenols. Currently, there are no studies in *B. bruxellensis* which explains the resistance mechanisms to hydroxycinnamic acids, and in particular to *p*-coumaric acid which is directly involved in alterations to wine. In this work, we performed a transcriptome analysis of *B. bruxellensis* LAMAP248rown in the presence and absence of *p-*coumaric acid during *lag* phase. Because of reported genetic variability among *B. bruxellensis* strains, to complement *de novo* assembly of the transcripts, we used the high-quality genome of *B. bruxellensis* AWRI1499, as well as the draft genomes of strains CBS2499 and0 g LAMAP2480. The results from the transcriptome analysis allowed us to propose a model in which the entrance of *p*-coumaric acid to the cell generates a generalized stress condition, in which the expression of proton pump and efflux of toxic compounds are induced. In addition, these mechanisms could be involved in the outflux of nitrogen compounds, such as amino acids, decreasing the overall concentration and triggering the expression of nitrogen metabolism genes.

*Brettanomyces bruxellensis* is one of the main contaminant yeasts in wines, with the ability to metabolize hydroxycinnamic acids, that are naturally present in grapes, into volatile phenols^1^,^2^,^3^,^4^. Hydroxycinnamic acids are weak acids with lipophilic character, such as *p*-coumaric acid, ferulic acid, and caffeic acid, all of which are able to adversely affect the organoleptic qualities of the wine giving undesirable odors^2^. Inhibitory effects of ferulic acid has been described on *Pichia anomala*^5^ and *Debaromyces hansenni*^6^, where the inhibitory effect is related to the high polarity of the acids. In wine, a large number of hydroxycinnamic acids have a synergistic effect which could enhance the inhibitory power. However, *Brettanomyces* species can overcome this toxicity by converting these acids into volatile phenols. The formation of these compounds in *B. bruxellensis* is a result of the enzymatic processing of hydroxycinnamic acids by the action of two specific enzymes, cinnamate decarboxylase (CD) and vinylphenol reductase (VR)^1^,^7^,^8^.

The presence of hydroxycinnamic acids in the culture medium can inhibit the growth of a wide range of microorganisms. However, this inhibition depends on the acid concentration^5^,^9^,^10^,^11^,^12^ and the type of molecule, with *p*-coumaric and ferulic acids showing the greater degree of inhibition^5^,^6^,^9^,^13^,^14^. The concentrations that can inhibit *B. bruxellensis* growth have been previously studied^6^,^15^, as well as the mechanisms involved^16^,^17^,^18^. These are based primarily on the chemical nature of the side chain carboxylic group (R-COOH) and its antimicrobial activity is based on the effects of the form of undissociated acid, which depends on the pH of the medium and the pK_a_ of the weak acid. The undissociated acid is able to go through the cell membrane by simple diffusion and activates several ATP demanding mechanisms that jeopardise the cell survivance^19^,^20^,^21^. In *Saccharomyces cerevisiae*, adaptation to weak acids has been has been related to the expression of different genes, mainly *PMA1* and *PDR12*, together with *VMA1*, *PDR5*, *MSN2*, *HSP26* and *MSN4*^22^,^23^,^24^. The response to weak acids has been extensively characterized in *S. cerevisiae*, *Candida albicans* and *Zygosaccharomyces bailii*^19^,^25^,^26^,^27^. In contrast, for *B. bruxellensis* no studies have evaluated the genes that are associated with the resistance to weak acids, and how they are taking part in the metabolism of hydroxycinnamic acids.

Previous studies of our group have shown that *B. bruxellensis* LAMAP2480 has significant differences in its growth curve, as well as in the production levels of volatile phenols, when grown in the presence of 100 mg/L of *p*-coumaric. Growth experiments in synthetic and natural wine^28^, showed that LAMAP2480 has a shorter duration of the *lag* phase and increased production of 4-ethylphenol compared to another strain (LAMAP1359)^28^, suggesting that LAMAP2480 has a rapid adaptation response to medium with *p*-coumaric acid, with a mechanism that allows for efficient resistance to this compound. Similar observations have been made for *B. bruxellensis* strains grown in the presence of different hydroxycinnamic acids^13^. We have also shown that the presence of *p*-coumaric acid at a concentration of 100 mg/L has a positive effect on the growth rate for some isolates of *Brettanomyces* spp^28^. The exposed antecedents suggests that the effect of this acid on the growth of this organism would be strain-dependent. This evidence suggests that the mechanism of weak acid resistance by *B. bruxellensis*, particularly to *p*-coumaric acid, is strain-dependent phenomenon that occurs during the *lag* phase of the growth curve.

*B. bruxellensis* has been poorly studied at the genetic level, and due to its genetic variability it has been a problem to develop models that describe its behaviour^29^,^30^,^31^,^32^,^33^,^34^,^35^. In some *B. bruxellensis* strains, the number of chromosomes can vary between 4 to 9^36^, with chromosome sizes in the range of 1 to 6 Mb, and a total genome size between 20 to 30 Mb^33^. *B. bruxellensis* karyotypic variation suggests speciation due to genome rearrangements. To this date, six *B. bruxellensis* strains from different sources (beer and wine) and geographical areas have been sequenced: AWRI1499^34^, CBS2499^35^, AWRI1608 and AWRI1613^37^, ST05.12/22^31^ and LAMAP2480^31^. Comparative genomic analysis of *B. bruxellensis* strains, showed that while CBS2499 is diploid, AWRI1499 and AWRI1608 are triploid, with two closely related alleles and a more divergent third one^37^. These findings suggested that some *B. bruxellensis* strains originate from three haplotypes. Preliminary evidence suggests that LAMAP2480 is triploid, and with the current draft state of its genome, this could introduce biases if only this genome is used as a reference for transcriptomic analysis. To complement this, and evaluate the effects of weak acids during *lag* phase in *B. bruxellensis*, we used two complementary approaches to study transcription profiles: *de novo* and genome guided transcriptome assembly.

## Results and Discussion

### Physiological evaluation of LAMAP2480 strain growth in *p*-coumaric acid

**Figure 1:**
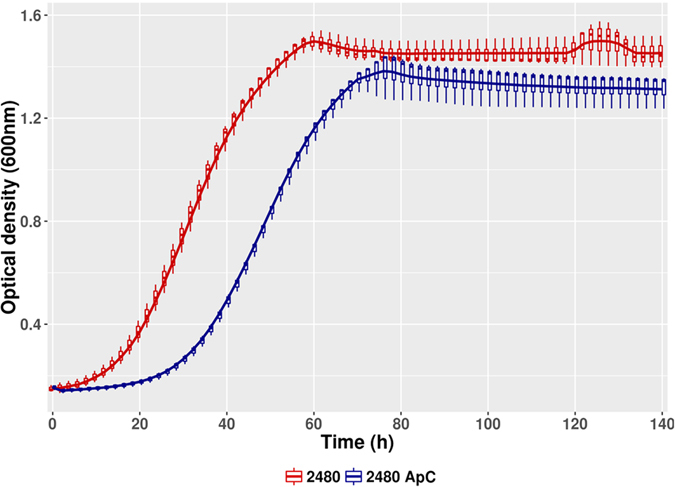
Cell growth *B. bruxellensis* LAMAP2480 as measured by OD600 for *p*-coumaric acid treated condition (blue line) and control (red line).

The presence of 100 mg/L *p*-coumaric acid in the culture medium caused an increase in the duration of the *lag* phase in LAMAP2480 (Fig. 1) from 14 hours (control) to 28 hours. Furthermore, we observed a reduction in the specific growth rate (μ) (Table 1) in presence of *p*-coumaric acid, which agrees with previous results^16^,^38^.

Measurements of extracellular pH during the *lag* phase showed a strong decrease in extracellular pH when LAMAP2480 was grown in the presence of *p*-coumaric acid, compared to the control condition. However, it has been reported that production of organic acids, such acetic acid, during yeast growth may also contribute to the process of extracellular acidification. Nevertheless, we previously demonstrated using High Performance Liquid Chromatography (HPLC) that acetic acid production starts during the exponential phase^39^,^40^. This suggests that the decrease of the culture pH was due to the action of the proton pump ATP-ase Pma1^41^.

**Figure 2:**
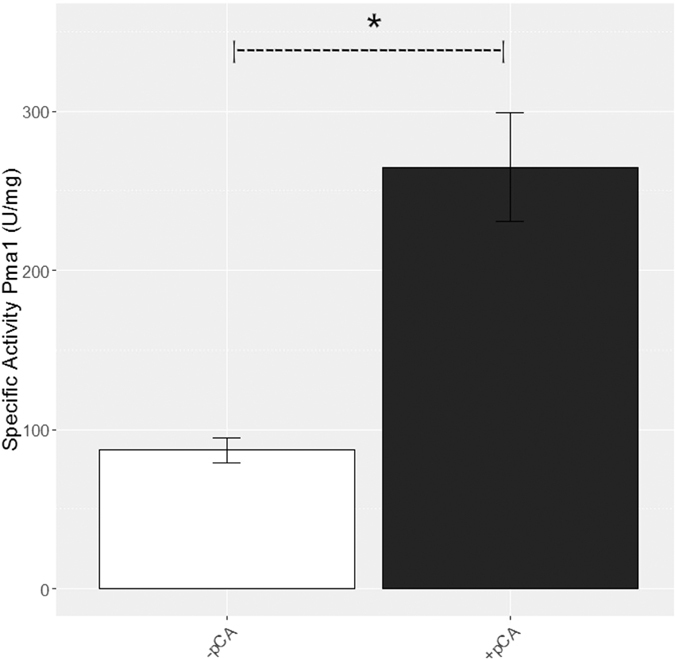
Pma1p specific activity during *lag* phase in *B. bruxellensis* LAMAP2480 grown in triplicates for control media (open column) and treatment media (*p*-coumaric acid at 100 mg/L) (black column). Statistical differences were evaluated using the student-t test with a level of significance of p < 0.05.

Pma1p activity was determined using protein extracts from cultures grown in Synthetic Dextose medium supplemented with *p*-coumaric acid during *lag* phase, and a higher activity was found in media containing *p*-coumaric acid (Fig. 2). This is similar to what has been previously reported for *B. bruxellensis* LAMAP1359 when grown in the presence of *p*-coumaric acid^14^. In the presence of weak acids, the role of Pma1p is vital to restore homeostasis^20^,^42^,^43^, which exerts a high energy demand, consuming until 60% of the total cellular ATP^20^, causing ATP levels to reduce to a point where growth rate declines^20^,^44^,^45^. Thus, the increase in the specific activity of Pma1p during *lag* phase in the medium supplemented with *p*-coumaric acid suggests that this proton pump is an important component of an adaptive response to hydroxycinnamic acids.

### Transcriptome analysis of *B. bruxellensis* LAMAP2480

Triplicates of two pools of mRNA samples were used to build libraries for RNA sequencing, generating approximately 197.7 millions of high quality paired-end reads, with an average length of 90 bp and encompassing 17,792 million nucleotides. The clean reads are available at the Sequence Read Archive (SRA) under accession number SRP077865.

Due to genomic differences between *B. bruxellensis* strains^31^,^32^,^33^,^34^,^35^,^36^,^46^, we performed transcriptional analysis using two approaches: *de novo* and reference-guided. To obtain a *de novo* transcriptome in which we could compare the abundance of transcripts against the genome-guided approach, all high quality reads were assembled into contigs using Trinity^47^. The assembly resulted in 26,761 transcripts with an average length of 1,570 base pairs, which were used to predict open reading frames (ORFs) and generated 23,650 non-redundant coding sequences, denominated Unigenes (Table 2).

**Figure 3:**
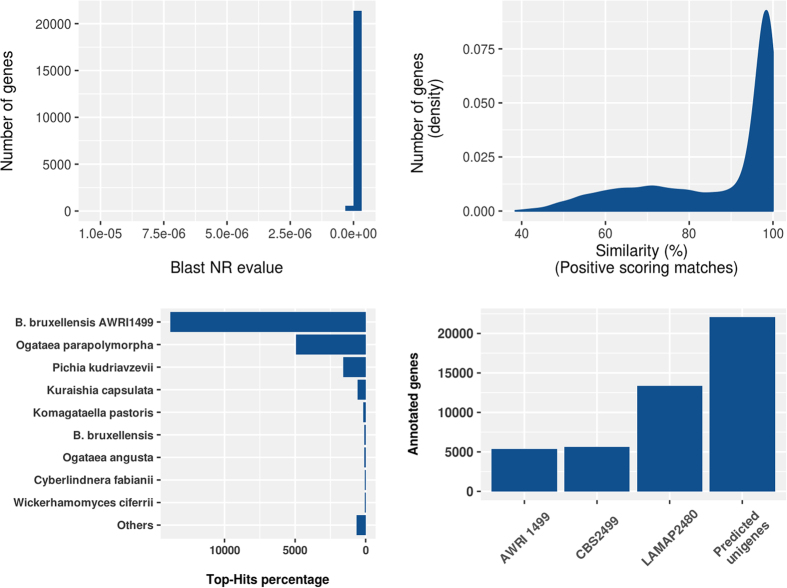
Characteristics of homology search of *B. bruxellensis* LAMAP2480 transcriptome unigenes with annotation to the NR database. (**A**) E-value distribution of annotated unigenes; (**B**) Similarity distribution of annotated unigenes (**C**) Number of unigenes matching the top 10 species. (**D**) Comparison of number of unigenes with existing *B. bruxellensis* strains.

In order to obtain the most complete annotated set of transcripts, the total of Unigenes were compared against the NCBI non-redundant database (NR), using Blastx. Approximately 93.3% (22,065 unigenes) were annotated with high homology, with an e-value ≤10^−5^ (Fig. 3A). Out of this total, a 70% of the genes were annotated with a sequence similarity greater than 80% (Fig. 3B). These results provide transcript information for genes expressed during *lag* phase of LAMAP2480 in SD media with presence of *p-*coumaric acid that were used for transcriptional downstream analysis.

As part of the annotation process, we evaluated the taxonomic distribution of the Blastx results for the *B. bruxellensis de novo* transcripts. As expected, the most represented yeast species was *B. bruxellensis* AWRI1499 (Fig. 3C) with 13,834 assignations, followed by *Ogataea parapolymorpha*, *Pichia kudriavzevii* and others. Although *B. bruxellensis* AWRI1499 is considered to be the best annotated strain, the number of contigs exhibiting homology with AWRI1499 only represents 62.7% of annotations (51.9% of the total of unigenes). These results strongly suggest that *de novo* assembly, complemented with *de novo* annotation of transcripts, can capture LAMAP2480 genes that are not well-annotated in the reference strain, as it was observed previously in *Schizosaccharomyces pombe*^47^.

The total number of *de novo* hits (22,065 unigenes) is an improvement compared to the existing gene annotations reported for AWRI1499 (4,861 genes), CBS2499 (5,650 genes) and LAMAP2480 (9,008 genes) (Fig. 3D), because we cover a higher number of transcripts that were not included in previous annotations. However, a single unigene represents the collection of expressed sequences that match a common transcript in a determined locus. These results must be interpreted carefully, because if two contigs match the same gene, it will produce an overestimation of annotations. When the data is filtered by removing multiple unigenes matching common genes, only 6,314 are unique annotations. This shows that *de novo* and genome-guided are complementary approaches that can be used to overcome the lack of annotation information for the *B. bruxellensis* genomes.

**Figure 4:**
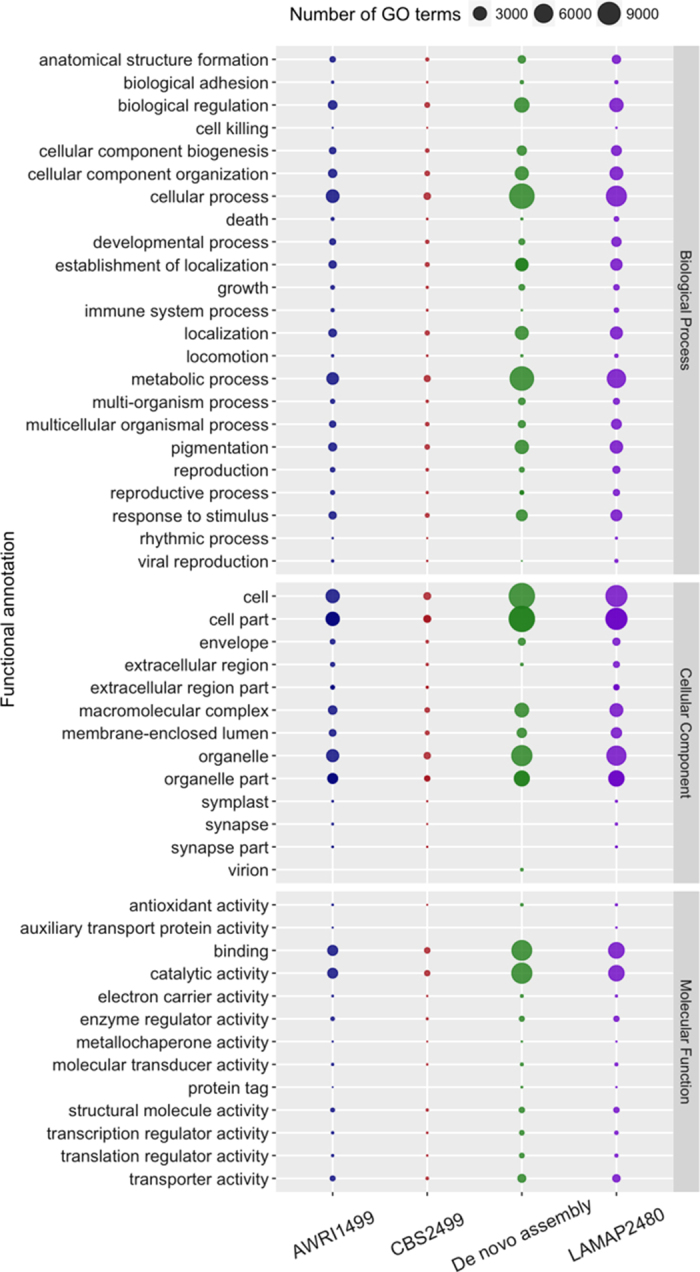
Comparative GO functional classification at second level of hierarchical GO tree from WEGO, for genes from *B. bruxellensis* AWRI1499, CBS2499, LAMAP2480 strains and unigenes annotated from *de novo* assembly. The results are summarized in the three main GO categories: Biological process, Cellular Component and Molecular Function.

None of *B. bruxellensis* genome annotations had Gene Ontology (GO) terms assigned, and GO terms assigned to *S. cerevisiae* did not covered the complete universe of annotated genes. To overcome this, we assigned GO annotation to all *B. bruxellensis* genomes and unigenes. Based on sequence homology of *B. bruxellesis* annotations, we annotated GO term to 3,432 AWRI1499 sequences, 3,779 CBS2499 sequences and 8,723 LAMAP2480 sequences. In the case of LAMAP2480 *de novo* assembled transcripts, a total of 16,054 sequences (72.76%) were annotated with GO terms. Sequence annotations against the GO database were performed using Blast2GO basic tool, and the data generated was stored in genome reference sessions known by the software as “work environments”, which file format has b2g extension. They can be used for enrichment analysis, KEGG pathways and are available for download (https://figshare.com/s/103665d0b83485b7e193). The distribution of functional categories for the three annotated genomes and the *de novo* transcriptome, was visualized looking at the higher hierarchical GO terms (Fig. 4). As expected, *de novo* assembly of LAMAP2480 transcripts generates a functional profile that is more similar to the genome annotation of this organism than to the other two strains (ARWI1499 and CBS2499). However, in the *de novo* transcripts we did not found categories such as biological process cell killing (GO:0001906) and rhythmic process (GO:0048511), and the molecular function auxiliary transport protein activity (GO:0015475), which were present in the genome annotation.

### Expression profiling during *p-coumaric* stress

To compare *de novo* assembly results with reference genomes, reads were aligned against the available genomes of *B. bruxellensis* strains using TopHat2^48^. As expected, the strain with a higher coverage was LAMAP2480 (91.78%), compared to strains AWRI1499 (62.22%) and CBS2499 (75.02%). Changes in gene expression produced by induction of *p-*coumaric stress were evaluated by normalizing *B. bruxellensis* gene expression levels using the RPKM method (Reads per kilobase transcriptome per million mapped reads). Principal component analysis (PCA) was used to plot the data of the control condition against the *p*-coumaric acid condition (Supplementary Fig. S1). The dispersion of the replicates indicates a high reproducibility per condition. Nevertheless, we observed that mapping against the CBS2499 genome did not show the same behavior on the replicates. Due to the low dispersion observed on the analysis with the other strains (AWRI1499 and LAMAP2480), we rule out that this distortion is due to technical problems. A possible source of variation for CBS2499 could be due to allelic variation, as CBS2499 has a diploid genome, while AWRI1499 is triploid, with a high degree of sequence divergence in one of the three alleles^37^. This evidence, combined with the low annotation frequency of *de novo* transcripts for CBS2499, may explain the distortion observed in the PCA plots, as part of the reads are not mapped to the genome.

**Figure 5:**
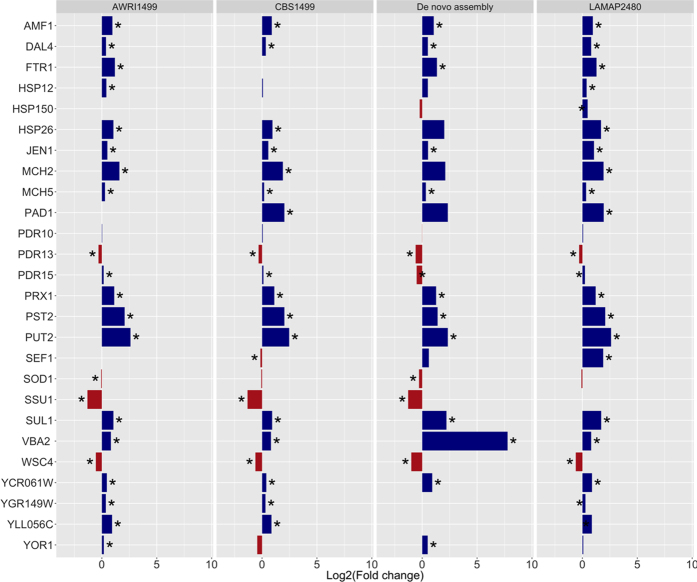
Gene expression pattern of genes related to *p*-coumaric acid response. Comparison per genome of Log2 fold-change expressed in RPKM for genes over (blue) or underexpressed (red) during *p-*coumaric treatment. Asterisk represents significance at corrected p-value <0.05.

Differentially expressed genes between the control and *p-*coumaric acid treatments were clustered based on the log_2_ fold-change and plotted against the p-value to observe overall gene expression patterns (Supplementary Fig. S2). Nevertheless, considering high genetic variability that exists between the genomes of *B. bruxellensis* strains, first we compared the expression of selected genes for validation related to the response to *p*-coumaric acid with each genome using log2 fold change expressed in RPKM (Fig. 5). The results, as expected, indicate that the expression patterns are different with each genome, however, pattern gene expression on *de novo* analysis and the strain LAMAP2480 are similar, and also in annotation and expression levels. Considering this, we used RNA-seq data generated at the draft genome LAMAP2480 as reference for validation. The results of gene expression in the figure revealed high expression differences between strains, which lead to validation of genes related to *p*-coumaric response.

### Validation of specific gene expression by RT-qPCR

**Figure 6:**
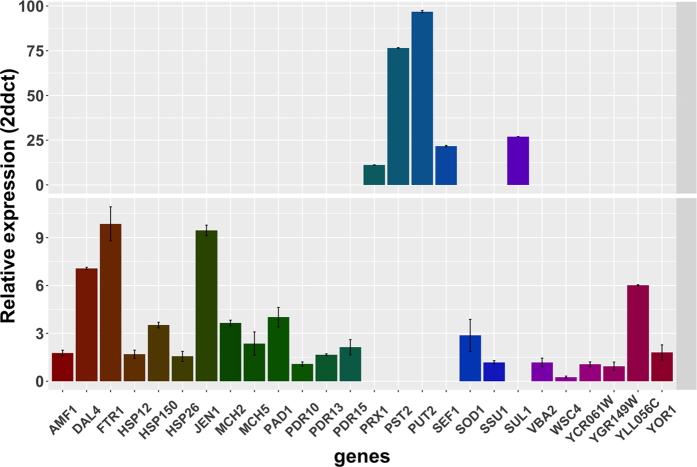
Gene expression during *lag* phase in LAMAP2480 strain determined by real-time PCR. Data (means ± SD) were calculated by the 2(-Delta Delta C(T)) method^104^ on three independent experimental replicates.

*B. bruxellensis* transcriptional response to stressing agents has been poorly studied^49^,^50^. In this study we evaluate *B. bruxellensis* LAMAP2480 response to *p-*coumaric acid stress during *lag* phase of growth curve. In this stage we observed differences of kinetic behaviour and we also found that in this stage *p*-coumaric acid is decarboxylated to produce 4-vinylphenol. Genes representative of functional categories or pathways that show a differential regulation by *p-*coumaric acid were selected for validation (Supplementary Table S1). Then, to validate the *p*-coumaric acid-induced transcriptional changes, qPCR was performed on three biological replicates per growth condition and relative expression was calculated (Fig. 6).

*PAD1* gene is overexpressed in the presence of *p*-coumaric acid. This gene codifies for a phenylacrylic acid decarboxylase^51^,^52^, and confers resistance to hydroxycinnamic acid by its decarboxylation to vinyl derivatives^51^,^53^,^54^. This gene was previously identified in LAMAP2480, and it was demonstrated that it plays a role in the decarboxylation of *p*-coumaric acid to 4-vinylphenol^52^. HPLC measurements shows that *p*-coumaric acid is decarboxylated at the start of the lag phase in the growth curve (Supplementary Fig. S3), as an early response mechanism to stress. *PAD1* is also present in CBS2499, but it has not been described for AWRI1499. To verify the presence of *PAD1* in AWRI1499, we aligned the *PAD1* known nucleotide sequence^52^ against its reference genome and we had a match in a non-annotated region (AHIQ01000324:33,529-34,042). This result allow us to complement the current reference annotation on AWRI1499 and may support future studies of decarboxylation in this strain.

*p*-coumaric acid induces the activation of transporters involved in efflux of toxic compounds and drug resistance (Supplementary Table S2). These transporters have been associated with the efflux of anions produced by the dissociation of weak acids^24^. Although it has been demonstrated that *p*-coumaric acid can be metabolized to less toxic compounds, such as 4-ethylphenol, the presence of transporters for *p-*coumaric acid or other derivatives has not been proved^1^,^24^. One possibility is that these transporters are responsible for the efflux of cumarate and/or its derivates. To evaluate this, the genes related to detoxification of toxic compounds and transports of drugs were studied. We observed overexpression of the genes *PDR13*, *PDR15*, *YLL056C*, *AMF1*, *YOR1*, and *PDR10*. These genes have been previously described to respond to the presence of propionic acid^55^, sorbic acid^23^,^55^, 2,4-D^56^,^57^ and Neocarzinostatin^58^. The resistance mechanism to this type of molecules involves ATP powered pumps, whose action can contribute to decrease the ATP levels in the cell. In the case of the gene *YLL056C*, it encodes for a putative protein of unknown function, and its transcription has been previously associated to response to the transcription factors Yrm1p and Yrr1p, as well as genes involved in pleiotropic drug resistance (PDR)^59^. In addition, it has been observed that *YLL056C* expression is induced in cells treated with different drugs^60^, such as the mycotoxin patulin^61^.

The treatment with *p-*coumaric acid also induced the expression of genes related to sulfate (*SUL1*) and iron (*FTR1*, *SEF1*) uptake. *SUL1* encodes for a sulphate transporter, and its overexpression has been previously reported as a resistance mechanism to neomycin in *S. cerevisiae*^62^. *FTR1* encodes for an iron permease and it is up-regulated by weak acids, being associated with an overall decrease in intracellular iron concentrations in *C. albicans*^60^. *SEF1* encodes for a putative transcription factor and has been related to be involved in controlling the expression of iron acquisition genes in *C. albicans*^59^. The overexpression of these genes suggest that the availability of cations is relevant to the resistance mechanism to weak acids, which is supported by previous evidence that the uptake of cations such as potassium, calcium and zinc are regulated in presence of propionic, acetic and lactic acids^55^,^63^,^64^.

In our experiment, genes that encode for monocarboxylate permeases (*MCH2* and *JEN1*) were overexpressed. These transporters are involved in the movement of monocarboxylic acids such as lactate, pyruvate, and acetate across the plasma membrane^65^,^66^. For *MCH2*, it has been previously reported that it is overexpressed in response to vanillin, a major phenolic compound generated due to lignin breakdown^67^, which suggest that it is involved in the transport of this compound^65^. It has also been observed that *JEN1* gene is induced by lactic and pyruvic acids^68^, which indicates that *p-*coumaric response is the induction of genes related to weak acids.

We also found that a gene-complex that is related to oxidative stress was regulated by the action *p*-coumaric acid. This complex is encoded by the genes *PST2* (flavodoxin-like protein), *PRX1* (mitochondrial peroxiredoxin) and *SOD1* (cytosolic copper-zinc superoxide dismutase). *PST2* is induced by oxidative stress in a Yap1p dependent manner and *PRX1* is induced during respiratory growth and oxidative stress^69^. It has been observed that *PST2* and *PRX1* genes are induced by oxidative stress in *S. cerevisiae*^67^,^70^ and *C. albicans*^66^. Correspondingly, it has been reported that *SOD1* is overexpressed in response to sorbic acid in *S. cerevisiae*^23^,^71^, suggesting that it plays a role in the resistance mechanism to ascorbic acid. Weak acids, such as sorbic, octanoic and decanoic acids has also been associated with the induction of multiple genes that take part in the response to oxidative stress. Because *p-*coumaric acid is also a weak acid, we can suggest that it induces a similar response and that the induction of *PST2* and *PRX1* are part of the resistance mechanisms of *B. bruxellensis*.

Under stress conditions mentioned above, the permeability of the cell wall is modified in order to reduce the rate of entry of weak acids to the cell^72^. Accordingly, we found genes involved in the synthesis of cell wall components that are DE in response to *p*-coumaric acid (Supplementary Table S2), such as chitin synthesis and cell wall assembly. We evaluated the expression of *WSC4* and *HSP150* by RT-qPCR. *WSC4* is involved in the translocation of soluble secretory proteins and the insertion of membrane proteins into the endoplasmatic reticulum membrane, while *HSP150* encodes for a O-mannosylated heat shock protein, that has been reported to be induced by heat shock, oxidative stress, and nitrogen limitation^73^. Our results indicate that *WSC4* is repressed in the presence of *p*-coumaric acid. While it has been reported that this gene is up-regulated after exposure to acetic acid by two-fold in *S. cerevisiae*^74^, other studies indicate that is repressed in response to the accumulation of misfolded proteins in the endoplasmic reticulum^75^. In contrast, *HSP150*, also annotated as *PIR2*, is overexpressed in the presence of *p*-coumaric acid. Previous reports have shown that this gene is overexpressed under stress in response to lactic and acetic acids^76^. Overall, the action of proteins encoded by both identified genes help restrict access of the *p*-coumaric molecules to the cell membrane.

In our analysis, a large number of genes associated with the use of nitrogen sources are highly represented (Supplementary Table S2). A first group is constituted of genes encoding for allantoin transporters and amino acid transporters, while a second group are genes that codify for enzymes involved in the metabolism of these compounds. *VBA2*, *PUT2*, and *DAL4* are overexpressed in the presence of *p-*coumaric acid, and were validated by qPCR. *VBA2* encodes for a protein involved in vacuolar uptake of basic amino acids^77^,^78^. *DAL4* encodes for a protein that takes part in the in the uptake of allantoin, a nitrogen-rich molecule generated from purine catabolism, which can be used as a nitrogen source by yeast cells^79^. *PUT2* encodes for a delta-1-pyrroline-5-carboxylate dehydrogenase^80^. *p*-coumaric acid also induced *MCH5*, which encodes for riboflavin transporter^81^, which has been reported to be highly expressed on proline medium^82^ and overexpressed during the *lag* phase of cells treated with 5-hydroxymethylfurfural (HMF)^83^. While the relationship between the toxicity of *p*-coumaric acid and an increase in the expression of these genes does not appear clear, it has been proposed in *S. cerevisiae* that an efflux pump that confers resistance to weak acids, can also carry amino acids outside the cell^84^. Furthermore, it has been previously reported that under stress conditions *S. cerevisiae* and *Arabidopsis thaliana* accumulate proline^80^, which is defense mechanism to create a nitrogen storage required for protein biosynthesis that in consequence acts as a protective agent for cells under osmotic stress^83^. Also, the upregulation of genes related to uptake and biosynthesis of amino acids has been observed in cells cultivated in the presence of acetic acid^85^,^86^, propionic acid^55^ and 2,4-D^57^. Supporting this, we found that the the gene *SSU1*, which encodes for a sulfite/nitrite membrane pump, was overexpressed in the presence of *p*-coumaric acid, and validated by qPCR. This pump exports excess sulfite produced during sulfate assimilation and amino acid biosynthesis^87^,^88^, suggesting that in the presence of *p*-coumaric acid, MFS transporters related to cellular detoxification also allow the output of important sources of nitrogen, leading to the depletion of intracellular free nitrogen, and inducing an increase in the expression of genes involved with the uptake and biosynthesis of amino acid, helping to maintain the balance the amino acid pool within cells. In addition, it has been reported that basic amino acid have a buffering capacity, which improves yeast growth by maintaining the pH close to neutral^89^. These amino acids can be stored in vacuoles using a cation-dependent transport mechanism, which could be related to the overexpression of cation permeases^90^.

Two heat shock proteins, *HSP12* and *HSP26*, were induced in the presence of *p*-coumaric acid. It has been previously reported that *HSP12* is overexpressed in yeast cells exposed to heat shock, as well as to osmotic and oxidative stress conditions^89^,^90^,^91^,^92^. In the case of *HSP26*, it has been associated with the ability to counteract HMF stress damage to proteins^83^. In yeast cells treated with sorbic acid, *HSP12* and *HSP26* were overexpressed^23^ conferring resistance to weak acid stress, which suggests this acid could be due to intracellular protein denaturation.

## Conclusion

We analyzed transcriptome data for *B. bruxellensis* LAMAP2480, for which prior genomic information was limited. The sequence information generated in this study allows to improve our understanding of the mechanisms that *B. bruxellensis* uses to respond to weak acid stress. In addition, this information restates the genetic differences between *B. bruxellensis* strains. Interpretations and accuracy of RNA-seq analysis results are highly dependent on the quality of a well-annotated reference genome. In this study we compared two draft genomes (LAMAP2480 and CBS2499), a complete reference genome (AWRI1499) and *de novo* assembly data. The results shows that when there is evidence of genetic variability among strains, *de novo* analysis complemented with a draft genome provides more accurate results than using a reference genome from a closely related strain.

**Figure 7:**
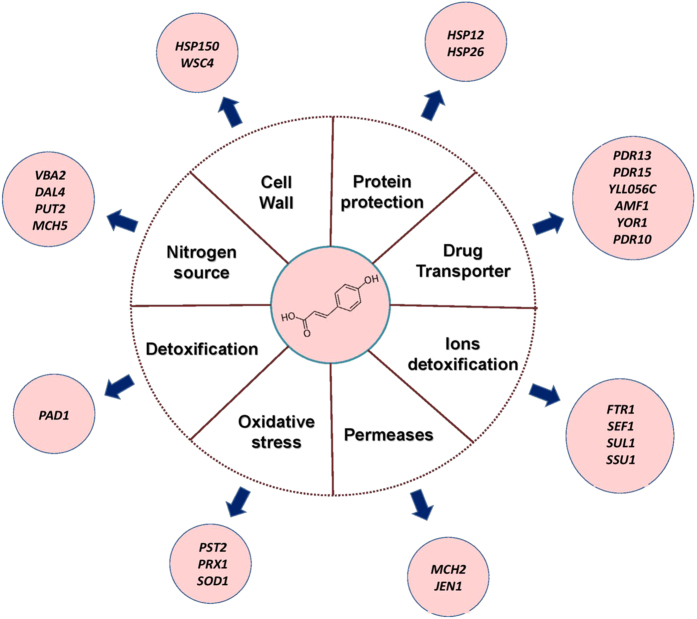
*B. bruxellensis* LAMAP2840 response to *p*-coumaric acid. A diagram of gene regulatory networks involving selective genes in yeast response to *p*-coumaric acid stress.

The results presented allow us to propose a model of early response to stress by *p*-coumaric acid. Its inflow into the cell causes a generalized stress condition, in which the expression of proton pumps and mechanisms involved in the efflux of toxic compounds are induced (Fig. 7). The latter might be involved in the outflow of nitrogen sources, such as as amino acids or allantoin, decreasing the intracellular concentration of nitrogen and triggering the expression of genes related to nitrogen metabolism. *p-*coumaric acid also induces oxidative stress, activating the expression of genes as *SOD1*, *PST2* and *PRX1*. Finally, the presence of this acid seems to cause protein denaturation, which is observed in the expression of chaperone proteins. At last this acid triggers a change of the permeability of the cell wall in order to reduce the rate of entry of weak acid into the cell.

## Materials and Methods

### Strain and culture media

*Brettanomyces bruxellensis* LAMAP2480 was originally isolated from Cabernet Sauvignon wine, and is part of the collection at the Laboratorio de Biotecnología y Microbiología Aplicada (LAMAP), Universidad de Santiago de Chile.

*P-coumaric* effect on *B. bruxellensis* LAMAP2480 growth was evaluated according to previous protocols developed in our laboratory with slight modifications^14^. The kinetic parameters evaluated were: specific growth rate (μ)^93^, *Lag* phase duration^94^, and efficiency was defined as area under curve (AUC) and expressed as a percentage using as 100% the control condition. Finally, growth inhibition (GI) was calculated as the percentage of the maximal specific growth rate of non-stressed control cultures^95^.

### Evaluation of physiological parameters

In order to describe the response of *B. bruxellensis* LAMAP2480 to *p*-coumaric stress, we evaluated seven physiological parameters: extracelullar pH, *p*-coumaric acid, 4-vinylphenol, 4-ethylphenol, acetic acid and glucose levels, and Pma1 enzyme activity. Extracellular pH was evaluated using previously described methods^22^, with adaptations to our local conditions and culture media^14^. *p*-coumaric acid, 4-vinylphenol, 4-ethylphenol, acetic acid and glucose were quantified by high performance liquid chromatography (HPLC) (Shimadzu Scientific Instruments, Colombia, MD, USA) using a Shimazdu Shim-Pack VP-ODS column^96^ for *p*-coumaric acid, 4-vinylphenol and 4-ethylphenol, and a Bio-Rad HPX87H column^97^ for acetic acid and glucose. Finally, plasma membrane ATPase Pma1p activity was estimated by calculating the rate of phosphate production after ATP hydrolysis^98^, as previously described^14^.

### RNA isolation, library preparation and Illumina sequencing

Cells were harvested after consumption of 10% of the glucose present in the medium, centrifuged at 2,850 g for 10 min and then resuspended in 200 μL RNA buffer (50 mM Tris-HCl pH 7.4; 100 mM NaCl; 10 mM EDTA) and 400 μL acidic phenol (pH 4.3). Cells were broken using acid-washed glass beads for 3 min with incubation on ice every 1 min. RNA buffer (200 μL) and 10% SDS (40 μL) were added and the mix was incubated for 6 min on 65 °C followed by centrifugation at 16,060 *g* for 15 min. Acidic phenol (400 μL) and 3 M NaOAc (40 μL) were added to the water phase^99^. After centrifugation at 16,060 *g* for 15 min, 1 mL of 96% ethanol was added and the mix was incubated 2 h. at −80 °C. The precipitated RNA was spun down at 4 °C for 10 min and was purified using the RNA Clean & Concentrator Column (Zymo Research). Total RNA was treated with DNase I (Promega, Madison, WI, USA). RNA quality was analyzed by CE-LIF (AATI Fragment Analyzer, Advanced Analytical Technologies Inc) to evaluate the RQN (RNA Quality Number.) All RNA samples had RNA Quality Numbers (RQNs) greater than 7.5.

RNA-seq library preparation and sequencing was carried out by the Beijing Genomics Institute (BGI) (Hong-Kong, China). Libraries were sequenced using Illumina HiSeq^TM^ 2000 (Illumina Inc, San Diego, CA, USA) in paired-end mode with a read length of 100 bp. The raw reads were cleaned by removing adaptor sequences and low quality sequences (q < 30).

### *De novo* and genome-guided RNA-seq analysis

With the aim of comparing gene expression of *B. bruxellensis* LAMAP2480 among strains, and evaluate differences between existing reference genomes, two different strategies were carried out; 1) *De novo* assembly and posterior annotation with Blast, 2) genome-guided mapping, aligning the raw reads to three *B. bruxellensis* strains: AWRI1499^34^, CBS2499^33^, and LAMAP2480^31^.

In undertaking the first analysis, reads were assembled into transcripts using Trinity, as previously described for de novo transcriptome assembly without a reference genome^47^. Transdecoder, an external Trinity plugin, was used to identify predicted open reading frames (ORFs) within isoforms or contigs and clustered into non-redundant sequences with a minimum length of 200 base pairs, which are known as unigenes.

Annotation was performed using NCBI BLAST 2.2.28+ with a minimum e-value cutoff of 1e-5 for the NCBI non-redundant protein (NR) database. Blast2GO^100^ software was used for gene ontology (GO) annotation in genomes from both approaches. Posteriorly, WEGO^101^ was used to perform GO functional classification at the second GO hierarchical level.

For gene expression analysis, reads mapped were normalized to RPKM (Reads per Kilobase per Million mapped reads) and edgeR^102^ was used for differential of expression. Genes differentially expressed were cut-off at significant level of 0.001 (p-values were adjusted with Benjamini & Hochberg method).

For the second approach, three *B. bruxellensis* strains genome and gene information were downloaded from NCBI. Alignment of reads to AWRI1499^34^, CBS 2499^33^ and LAMAP2480^31^ was performed using TopHat2^48^. To improve accuracy of differential expression analysis, samples were merged before analysis with Cufflinks, improving with this the insufficient depth of coverage for genes with low expression values, as described in Tuxedo protocol^103^. For the differential expression analysis a gene was considered to have significant changes with FDR < 0.05.

### Validation of differentially expressed genes by real-time PCR

Quantitative RT-PCR analysis was used to validate the expression of the candidate genes. RT-qPCR was conducted on Applied Biosystems StepOnePlus Real-Time PCR System using StepOne Software (v2.0) (Applied Biosystems, Foster City, CA, USA) according to the manufacturer’s instructions. All qRT PCR primers were designed using qRT primer design tools available online (idtdna.com), and designed to amplify fragments between 150 and 250 base pairs. All qRT-PCR reactions were run in 20 ul reactions using using 5x HOT FIREPol^®^ EvaGreen^®^ qPCR Mix Plus (ROX) (Solis BioDyne, Tartu, Estonia) according to the SYBR Green method. Each reaction contained 4 μL 5x EvaGreen^®^ qPCR Mix Plus (ROX), 1 μL primer mix (250 nM each), 1 μL cDNA (1 ng), and 13 μL DNase/RNase free water. Amplification was carried out with the following cycling parameters: heating for 15 min at 95 °C, 40 cycles of denaturation at 95 °C for 15 s, annealing at 58 °C for 20 s and extension at 72 °C for 20 s. Each sample was analyzed in triplicates and the expression values were normalized against β−actin. The molecular weight of the products was confirmed via diagnostic agarose gel and the melting curves were analyzed. Analysis of the relative gene expression data was conducted using the 2^−ΔΔ*C*T^ method^104^.

## Additional Information

**How to cite this article**: Godoy, L. *et al.* Comparative transcriptome assembly and genome-guided profiling for *Brettanomyces bruxellensis* LAMAP2480 during *p-coumaric* acid stress. *Sci. Rep.* **6**, 34304; doi: 10.1038/srep34304 (2016).

## Supplementary Material

Supplementary Information

Supplementary Table S1

Supplementary Table S2

## Figures and Tables

**Table 1:** Kinetic parameters of growth *B. bruxelllensis* LAMAP2480.

	Parameter				
						μ (h^−1^)	*lag* (h)*	T*g* (h)	Efficiency (%)¥	GI (%)
LAMAP2480	0.072^a^ ± 0.001	14.094 ± 1.089	9.63	100	
LAMAP2480 *p*CA	0.065^b^ ± 0.001	28.188 ± 1.789	10.66	79	9.8

^*^Buchanan y Cygnarowicz 96.

^¥^Defined as area under curve and expressed as a percentage using as 100% the control condition; Growth inhibition.

**Table 2:** Summary of *de novo* assembly of transcriptome sequence reads without using *B. bruxellensis* reference genome.

	Sequences/Contigs	Base Pairs	Average Length	N50	GC percentage
Reads	98846216	98846216	90	—	—
Transcripts	26761	42024611	1570.37	2483	44.41
Unigenes	23650	29604087	1251.76	1557	44.11
