# Supplementary material for: Comparative transcriptome assembly and genome-guided profiling for Brettanomyces bruxellensis LAMAP2480 during p-coumaric acid stress
Source: Sci Rep. 2016 Sep 28;6:34304. doi: 10.1038/srep34304 (PMC5039629; doi:10.1038/srep34304)
Supplement: Supplementary Information [file srep34304-s1.pdf]

Comparative transcriptome assembly and genome-guided profiling for *Brettanomyces bruxellensis* LAMAP2480 during *p-coumaric* acid stress

Liliana Godoy[1+], Patricia Vera-Wolf[1,2+], Claudio Martinez[1,3], Juan A. Ugalde[2], Angélica Ganga[1,4*]

[1] Laboratorio de Microbiología Aplicada y Biotecnología, Departamento en Ciencia y Tecnología de los Alimentos, Facultad Tecnológica, Universidad de Santiago de Chile, Santiago de Chile, Chile

[2] Centro de Genética y Genómica, Facultad de Medicina, Clínica Alemana Universidad del Desarrollo.

[3] Centro de Estudios en Ciencia y Tecnología de Alimentos, Universidad de Santiago de Chile.

[4] Millennium Nucleus for Fungal Integrative and Synthetic Biology (MN-FISB) 120043

**Corresponding author*

E-mail: angelica.ganga@usach.cl (Angélica Ganga)

[+]these authors contributed equally to this work

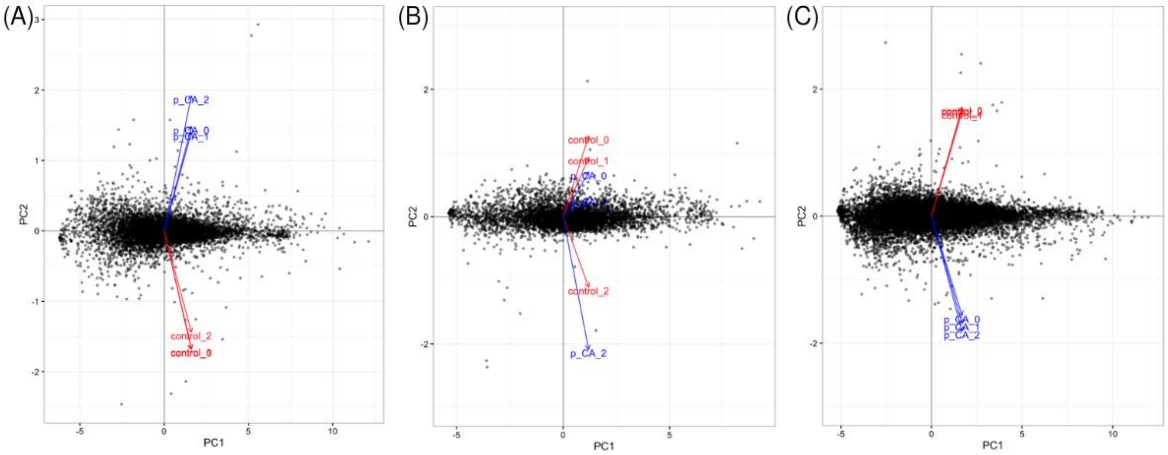

Figure S1. Principal component analysis (PCA) of *B. bruxellensis* LAMAP2480 transcripts in presence and absence of *p*-coumaric acid. Reads from RNA-seq analysis mapped against three *B. bruxellensis* reference genomes: (A) AWRI1499, (B) CBS2499 and (C) LAMAP2480. Red samples represents control triplicates and blue samples shows treated triplicates with *p*-coumaric acid.

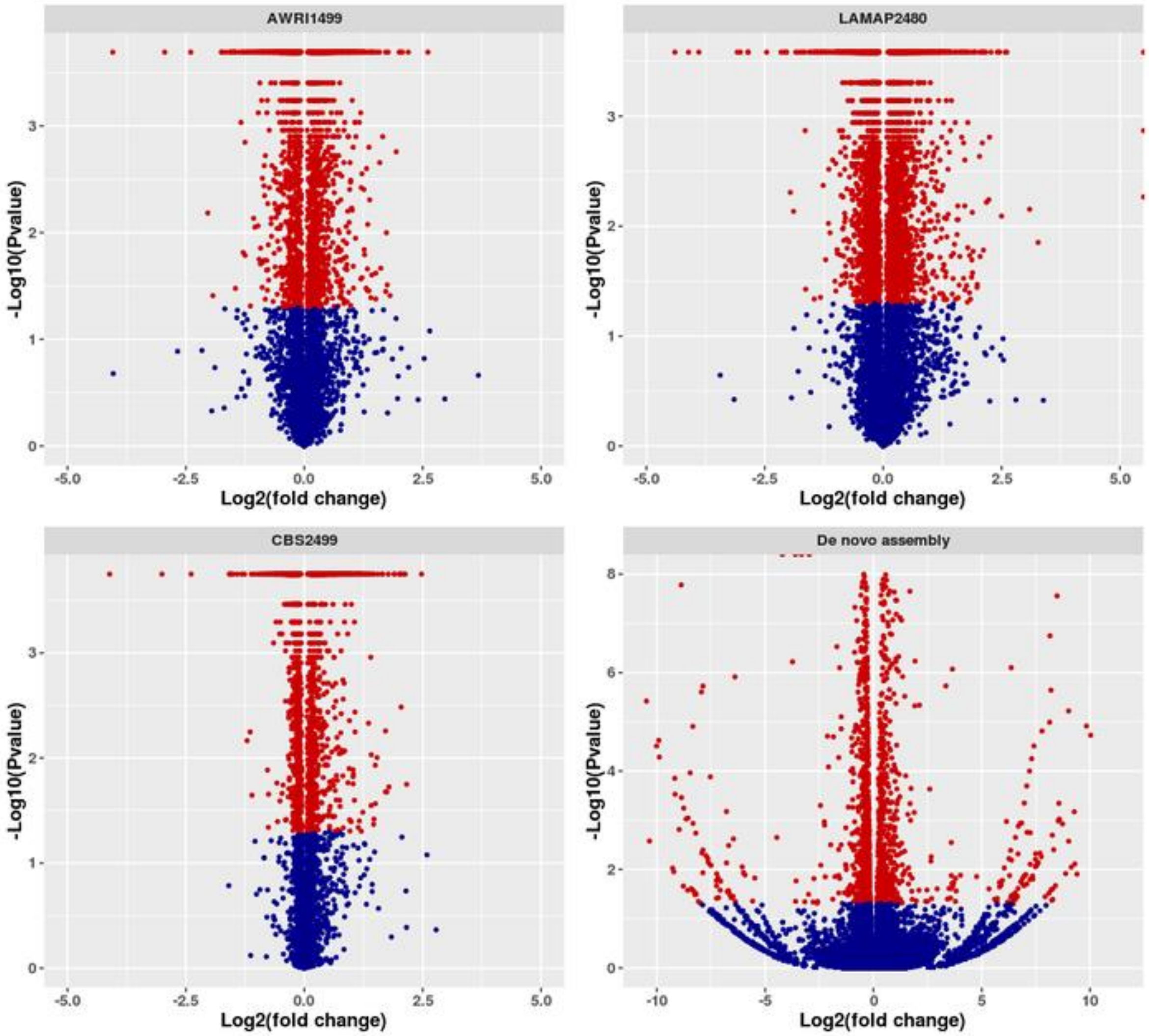

Figure S2. Volcano plot illustrating *B. bruxellensis* genomic differences at transcription level between *p*-coumaric acid treatment and control conditions. Positive values represent upregulated genes, negative downregulated genes and DEG were filtered by significance of corrected P values < 0.05 shown as red dots.

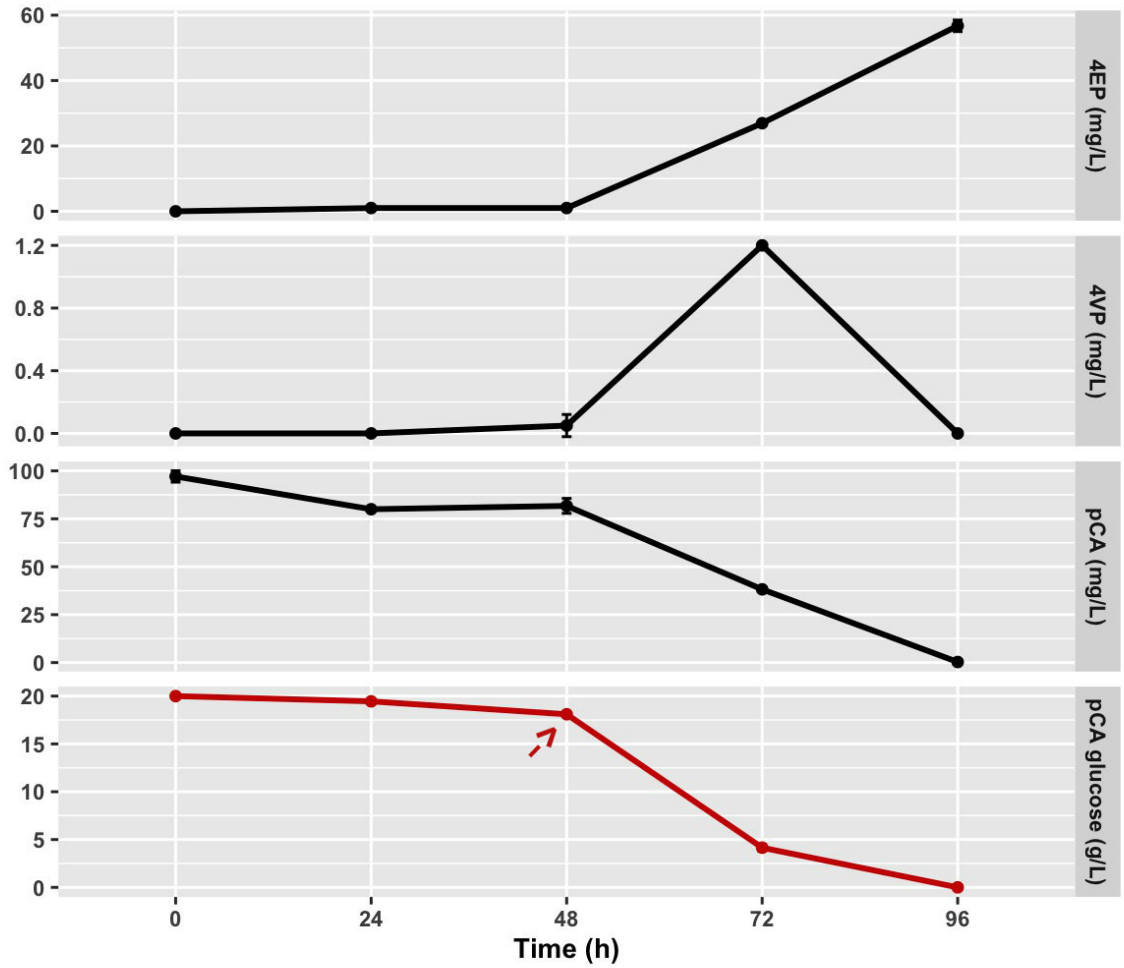

Figure S3. Kinetic production of 4-ethylphenol (4EP) and 4-vinylphenol (4VP) and consumption of *p*-coumaric acid (pCA) and glucose (pCA glucose) during *B. bruxellensis* LAMAP2480 growth in pCA treatment condition. Red arrow represents the point in which samples for RNAseq experiment were collected.
